# Screening of rosmarinic acid from *Salvia miltiorrhizae* acting on the novel target TRPC1 based on the ‘homology modelling–virtual screening–molecular docking–affinity assay–activity evaluation’ method

**DOI:** 10.1080/13880209.2022.2160769

**Published:** 2023-01-05

**Authors:** Wei Quan, Yuan Wang, Yu-han Chen, Qing Shao, Yang-ze Gong, Jie-wen Hu, Wei-hai Liu, Zi-jun Wu, Jie Wang, Shan-bo Ma, Xiao-qiang Li

**Affiliations:** a Department of Pharmacy, Affiliated Hospital of Shaanxi University of Chinese Medicine, Xianyang, China; b Department of Pharmacology, School of Pharmacy, Air Force Medical University, Xi’an, China; c Department of Neurosurgery, Wuhan No.1 Hospital, Wuhan, China; d Department of Pharmacy, Xijing Hospital, Air Force Medical University, Xi’an, China; e Xi’an Mental Health Center, School of Medicine, Xi’an Jiaotong University, Xi’an, China

**Keywords:** Transient receptor potential canonical channel 1, drug screening, myocardial injury

## Abstract

**Context:**

*Salvia miltiorrhizae* Bunge (Lamiaceae) is a traditional Chinese medicine (TCM) for the treatment of ‘thoracic obstruction’. Transient receptor potential canonical channel 1 (TRPC1) is a important target for myocardial injury treatment.

**Objective:**

This work screens the active component acting on TRPC1 from *Salvia miltiorrhizae*.

**Materials and methods:**

TCM Systems Pharmacology Database and Analysis Platform (TCMSP) was used to retrieve *Salvia miltiorrhiza* compounds for preliminary screening by referring to Lipinski’s rule of five. Then, the compound group was comprehensively scored by AutoDock Vina based on TRPC1 protein. Surface plasmon resonance (SPR) was used to determine the affinity of the optimal compound to TRPC1 protein. Western blot assay was carried out to observe the effect of the optimal compound on TRPC1 protein expression in HL-1 cells, and Fura-2/AM detection was carried out to observe the effect of the optimal compound on calcium influx in HEK293 cells.

**Results:**

Twenty compounds with relatively good characteristic parameters were determined from 202 compounds of *Salvia miltiorrhiza*. Rosmarinic acid (RosA) was obtained based on the molecular docking scoring function. RosA had a high binding affinity to TRPC1 protein (KD value = 1.27 µM). RosA (50 μM) could reduce the protein levels (417.1%) of TRPC1 after oxygen-glucose deprivation/reperfusion (OGD/R) in HL-1 cells and it could inhibit TRPC1-mediated Ca^2+^ influx injury (0.07 ΔRatio340/380) in HEK293 cells.

**Discussion and conclusions:**

We obtained the potential active component RosA acting on TRPC1 from *Salvia miltiorrhizae*, and we speculate that RosA may be a promising clinical candidate for myocardial injury therapy.

## Introduction

Transient receptor potential canonical channel (TRPC) is a kind of Ca^2+^-permeable non-selective cation channel. Based on the amino acid sequence, the TRPC family is divided into seven isoforms (TRPC1–7, among which TRPC2 is a human pseudogene) (Wang et al. 2020). TRPC is expressed in several organs, including the heart, brain and kidney. Store-operated calcium entry (SOCE), as one of the major pathways of calcium entry, plays an important role in intracellular calcium homeostasis, affecting important cellular processes (Prakriya and Lewis 2015). TRPC1 is also the main ion channel involved in SOCE (Dyrda et al. 2020), and the downregulation of TRPC1 reduces SOCE by approximately 60% (Ambudkar et al. 2017). Recent studies have shown that TRPC1 and Ca^2+^-related pathways mediated by it are involved in the regulation of many physiological and pathological processes, including neuronal survival (Sun et al. 2017), glomerular mesangial cell contraction (Du et al. 2007), skeletal muscle development (Antigny et al. 2017) and diabetes mellitus (Krout et al. 2017). Additionally, abnormal expression of TRPC1 may be involved in the progression of many cardiovascular system diseases, including myocardial hypertrophy (Inoue et al. 2006), vascular injury and generation (Martin-Bornez et al. 2020) and atherosclerosis (AS) (Hof et al. 2019). According to our experimental results, under the conditions of HL-1 oxygen-glucose deprivation/reperfusion (OGD/R), Ca^2+^ in cardiomyocytes was not significantly changed after the inhibition of voltage-dependent calcium channel (VDCC) with nifedipine. In contrast, Ca^2+^ influx was significantly reduced after the application of non-selective TRP channel blocker SKF-96365 or the *Trpc1* gene silencing. The above studies suggest that TRPC1 may be involved as an important target in myocardial injury.

Traditional Chinese medicine (TCM) has been used in China for thousands of years. Against the background of high cost, long period and difficulty of innovative drug research, searching active compounds from TCM has re-emerged as a hot topic in recent years (Chen et al. 2021). *Salvia miltiorrhizae* Bunge (Lamiaceae) is a commonly used traditional herb with an application history of nearly 2000 years. Exploring active components of *Salvia miltiorrhizae* and revealing their pharmacological effects are the key points of its modernization research (Zou et al. 2018). It has been reported that water decoction of *Astragali* (Fabaceae) and *Salvia miltiorrhizae* can inhibit isoprenaline (ISO)-induced myocardial remodelling of rats, and the inhibition mechanism is related to the downregulation of TPRC1 protein expression (Wang et al. 2017). *Astragalus membranaceus*–*Salvia miltiorrhizae* pills can regulate calcium homeostasis through the TRPC1/STIM1 pathway, promote the synthesis and release of vasodilation factor NO, and inhibit the inflammatory response, thus exerting anti-AS effects (Hu et al. 2019). In addition, tanshinone IIA, an active component of *Salvia miltiorrhizae*, can significantly inhibit *Trpc1* mRNA and protein expression, reduce SOCE opening, decrease intracellular Ca^2+^ concentration, and therefore inhibit the migration and spreading of pulmonary artery smooth muscle cells (Wang et al. 2013). All the above studies suggest that *Salvia miltiorrhizae* and its active components are closely related to the transcription and expression of TRPC1 molecules.

As an efficient method for the identification of lead compounds, molecular docking virtual screening technology has been widely used for drug discovery in various fields (Gruneberg et al. 2002). Many lead compounds acting on target proteins related to major diseases have been successfully identified using the molecular docking technique (Zhang et al. 2020). Recently, an article about the inhibitor of the Mpro (SARS-CoV-2 enzyme) of the 2019 novel coronavirus (2019-nCoV) was published in *Nature* (Jin et al. 2020). Through molecular docking virtual screening, the research team identified N3, a potent inhibitor of Mpro. Therefore, we adopted ‘homology modelling–virtual screening–molecular docking–affinity assay–activity evaluation’, a new TCM drug discovery and evaluation model, to screen the exact active component acting on TRPC1 from *Salvia miltiorrhizae*, a TCM that promotes blood circulation and dispels blood stasis. It is expected that this study will provide an entry point and lay the foundation for future exploration of multi-component and multi-target pharmacodynamic substances in TCM and their coordinated mechanism.

## Materials and methods

### Compounds of *Salvia miltiorrhizae*

TCMSP (Traditional Chinese Medicine Systems Pharmacology Database and Analysis Platform) is a unique systems pharmacology platform of Chinese herbal medicines (http://tcmspw.com/tcmsp.php). All compounds of *Salvia miltiorrhizae* reported in TCMSP were sorted.

### TRPC1 homology modelling

Since the crystal structure of TRPC1 protein was not recorded in the PDB database, we first performed TRPC1 homology modelling with the best template of TRPC1 obtained from Swiss-Model (http://swissmodel.expasy.org/). Then, a homologous 3D structure of TRPC1 protein was constructed, and its quality was evaluated with the Ramachandran plot.

### Kinetic optimization simulation of TRPC1

Molecular dynamics simulations were performed using Gromacs 2020.1, in which the charm36-jul2020 force field was chosen. The protein and molecule complex were solved with TIP3P water and immersed in a dodecahedron box extending to at least 1 nm of the solvent on all sides. Also, the system was neutralized by Na^+^ and Cl^–^, and then 0.15 M NaCl was added. The energy was minimized by the steepest descent algorithm for 5000 steps, and it generated a maximum force of less than 1000 kJ/mol/nm. After energy minimization, the system was equilibrated in a constrained NVT (number of particles, volume, temperature) and NPT (number of particles, pressure, temperature) and ran for 100 ps. NVT equilibration ensured the desired temperature (300 K), under which we sought to establish the proper orientation of the protein. After NVT equilibration, we stabilized the pressure of the system under an NPT ensemble. Through NVT and NPT equilibration, it was well-equilibrated at 300 K and 1 bar. Finally, MD simulations of the TRPC1 were carried out for 100 ns. Trajectories were saved every 10 ps for analysis. The Verlet cut-off scheme and a Leap-frog integrator with a step size of 2 fs were applied. For temperature coupling, the modified Berendsen thermostat and the Parrinello-Rahman barostat for pressure coupling were used. For long-range electrostatic interaction, the particle mesh Ewald method was used. The root-mean-square displacement (RMSD) was calculated by GROMACS 2020.1.

### Molecular docking and analytical mapping

The TRPC1 protein homology modelling structure was uploaded to POCASA (http://altair.sci.hokudai.ac.jp/g6/service/pocasa/) to predict the potential binding sites. Grid Box coordinates and box size were set according to these potential binding sites, and then AutoDock Vina 1.1.2 was applied for molecular docking. The parameters were set as: center_*x* = 113.0, center_*y* = 98.0, center_*z* = 57.0; size_*x* = 50, size_*y* = 50, size_*z* = 50. A total of nine conformations were generated, and the conformation with the best affinity was selected as the final docking conformation. Maestro 11.9 was used to plot the interactions.

### Cells

Cardiomyocyte cell line HL-1 and human embryonic kidney cell line HEK293 were provided by Shanghai Huzhen Biotechnology Co., Ltd. (Shanghai, China). Culture medium formula: 10% foetal bovine serum + 1% penicillin-streptomycin + DMEM. Culture condition: 37 °C, 5% CO_2_. The primary cells were subcultured in culture flasks with a split ratio of 1:4 and after about 2–3 days, the cells completed about 85% growth.

### Main materials and reagents

DMEM/low- and high-glucose medium (Hyclone, Logan, UT), GAPDH primers (Tsingke Biotechnology Co., Ltd., Beijing, China), HRP-labelled goat anti-rabbit IgG (Beijing ComWin Biotech Co., Ltd., Beijing, China), TRPC1 antibody (Alomone Labs, Jerusalem, Israel), TRPC1 protein fragment (Alomone Labs, Jerusalem, Israel), *Trpc1* primer (Tsingke Biotechnology Co., Ltd., Beijing, China), β-actin antibody (Sigma, St. Louis, MO), rapid qPCR mix (SYBR Green I) kit (Tsingke Biotechnology Co., Ltd., Beijing, China), reverse transcription RT6 cDNA synthesis kit (Tsingke Biotechnology Co., Ltd., Beijing, China), total RNA extraction kit (Tsingke Biotechnology Co., Ltd., Beijing, China), Fura-2/AM calcium fluorescent probe (Invitrogen, Carlsbad, CA), OAG (Cayman, Ann Arbor, MI), rosmarinic acid (RosA) (lot number: ITM10073169) (Shanghai Topscience Co., Ltd., Shanghai, China).

### SPR determination of compound–protein affinity

The surface plasmon resonance (SPR) instrument from Affinité Instruments (Montreal, Canada) was applied. After activating the gold membrane with NaAC buffer at pH = 5.0 for 10 min, the TRPC1 protein fragment was added to NaAC buffer at pH = 5.0 to configure a 250 nM solution. The configured solution was injected into the line and reacted for 15 min. Then, unbound fragments were rinsed with PBS. Binding assays were performed using RosA in different concentrations to determine the affinity. After each assay, the rinsing with PBS for 2 min, followed by the rinsing with 0.5% SDS solution for 2 min, was conducted.

### Construction of HL-1 cell OGD/R injury model

HL-1 cells were cultured in a DMEM medium (containing foetal bovine serum with a volume fraction of 10%) and placed in an incubator containing 5% CO_2_ at 37 °C. Cells at the logarithmic growth phase were selected for the experiment. HL-1 cells were inoculated onto a cell culture plate, and when they completed 85% growth, the original medium was poured off. After the rinsing with PBS, the DMEM medium was completely replaced by OGD solution (low-glucose DMEM medium containing sodium dithionite at a concentration of 2.5 mmol/L). The plate was then placed in a constant temperature (37 °C) incubator for 9 h. The plate was taken out, and the OGD solution was replaced by a high-glucose DMEM medium (containing foetal bovine serum with a volume fraction of 10%). After that, the plate was incubated in a cell incubator for OGR for 6 h. The control group (normoxia group) was placed in an incubator for 15 h after DMEM medium replacement.

### Western blot assay for HL-1 cells protein expression

The supernatant of the protein lysate solution was transferred into a new EP tube for immediate protein concentration determination and protein content standardization. Then, 10% separation gel and 5% concentrated gel were prepared. The loading amount of the protein sample was determined according to the standardized content. One well was left on the left and right sides. Then, 4 μL rainbow marker and 3 μL luminous marker were added. The voltage was set at 80 V. Thirty minutes after the electrophoresis of the sample to the separation gel, the voltage was increased to 120 V. The transfer buffer was prepared and pre-cooled in the refrigerator. The separation gel was carefully transferred to the NC membrane and they were clamped. The gel holder cassette was placed at the transfer tank. Then ice bath was performed under the voltage of 100 V for 100 min. The membrane was stained with ponceau S for 2 min and rinsed fully with PBST. The first antibody was diluted by 5% protein blocking buffer, which was prepared by skimmed milk powder plus PBST (TRPC1 dilution ratio 1:1000, endogenous reference β-actin dilution ratio 1:2000) and was shaken for 1 h at room temperature. The strips were incubated at 4 °C overnight. The second antibody was incubated (PBST dilution ratio 1:5000) by a shaker at room temperature for 1.5 h and rinsed three times with PBST. The chemiluminescence imaging system was used for photographing. The photos were analysed using Image-Pro Plus software to obtain strip grayscale values.

### Fura-2/AM detection for Ca^2+^ concentration in HEK293 cells

The preparation of Tyrode’s mother solution (10×): 3.680 g NaCl, 0.202 g KCl, 1.192 g HEPES, 0.102 g MgCl_2_·6H_2_O, 0.026 g NaH_2_PO_4_·2H_2_O were dissolved in 50 mL distilled water, mixed and stored at 4 °C. The preparation of calcium-free Tyrode’s solution: 50 mL of Tyrode’s mother solution (10×) was diluted to 500 mL with distilled water. Then, 1 g glucose and 2 mL EGTA solution with a concentration of 10 mM were added, mixed and stored at 4 °C. Fura-2/AM fluorescent dye was made into 1 mmol/L Fura-2/AM mother liquor by dissolving 50 µg Fura-2/AM powder with 49.9 µL dimethyl sulphoxide (DMSO). The mother liquor was divided, with each dividing tube containing 2.5 μL, and frozen at −20 °C. The liquor in the dividing tube was diluted 100 times with calcium-free Tyrode’s solution during the experiment. Then, 125 μL fresh medium was mixed with 125 μL Fura-2/AM in a well of a new 24-well plate. The cell-attached slide was placed into the well and set aside at room temperature without light for 30 min. After that, the cells were loaded with Fura-2/AM. The Fura-2/AM-loaded cell-attached slide was moved out and placed at the centre of the bath. Then, the slide was perfused with calcium-free Tyrode’s solution for 5 min to remove the excess dye on the cells. The peristaltic pump was turned off, and it was confirmed that there was enough calcium-free Tyrode’s solution in the bath to form an aqueous film with the microscope objective. The bath was fixed on the carrier of the inverted fluorescence microscope. The software was launched, and the timer was started. After 3 min, OAG (TRPC agonist) and CPA (calcium channel agonist modulated with internal calcium store) solution were added to the bath and reached a final concentration of 100 μΜ. After 5 min, 1.8 m M2 was added to the bath, and *F*_340_/*F*_380_ (i.e., fluorescence intensity ratio) was recorded to reflect the change in Ca^2+^.

### Data statistics and analysis

Statistical analysis of mean differences between the two groups was performed using Student’s two-tailed *t*-test. ANOVA was used for multiple comparisons between groups, and Tukey’s test was applied for comparison between groups. The statistical software was GraphPad Prism 8 (La Jolla, CA), and *p* < 0.05 indicated a statistically significant difference.

## Results

### TRPC1 homology modelling and model evaluation

**Figure 1.**
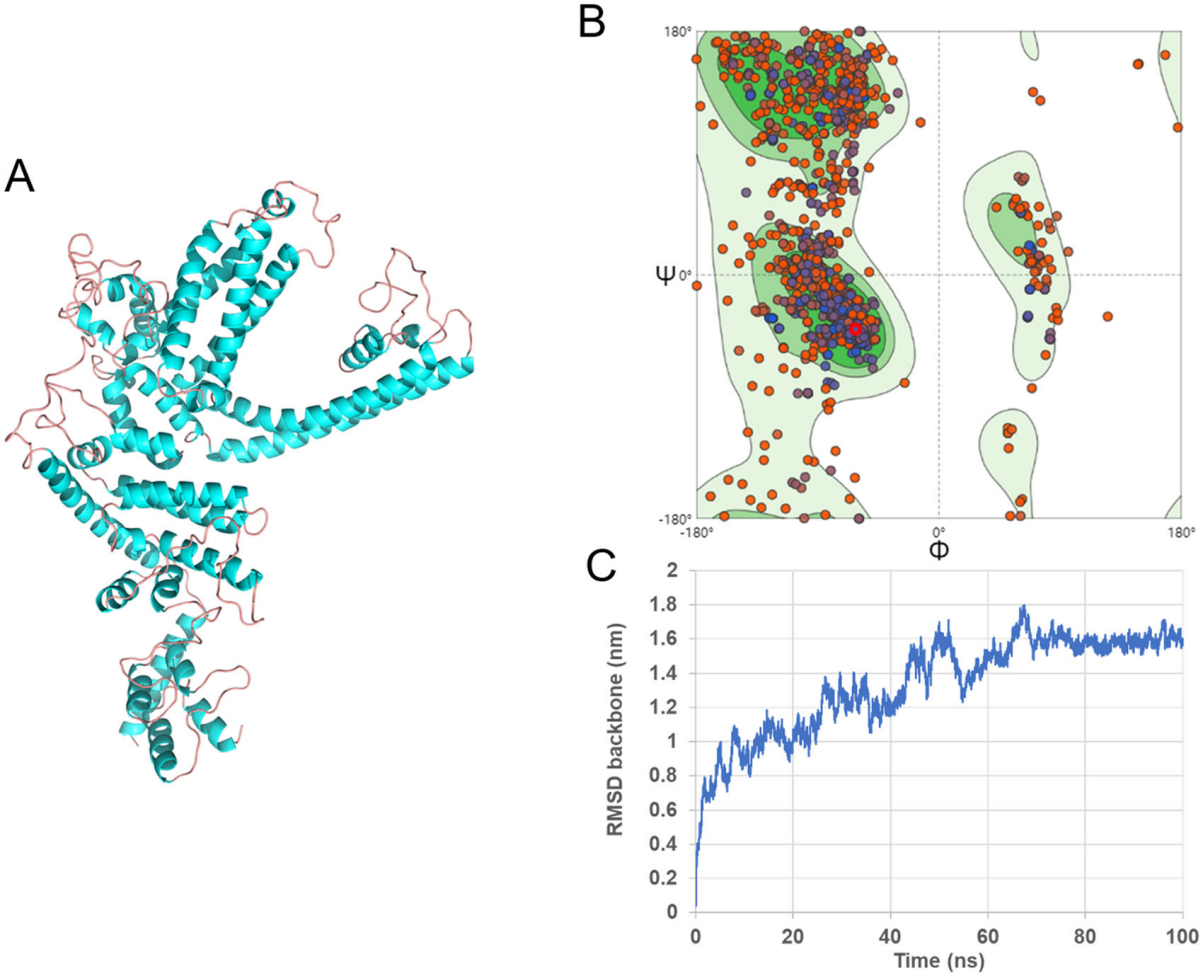
(A) Schematic diagram of the three-dimensional structure of TRPC1 protein. Blue represents α-helix, pink represents random curl. (B) Evaluate the three-dimensional structure of TRPC1 protein according to the Ramachandran plot. The green, light green and light blue regions represent the ‘preferred’, ‘allowed’ and ‘usually allowed’ regions that amino acid residue fall regions, respectively. (C) Molecular dynamics simulation was performed. A stable conformation of TRPC1 was randomly selected between 80 ns and 100 ns for subsequent docking experiments.

**Figure 2.**
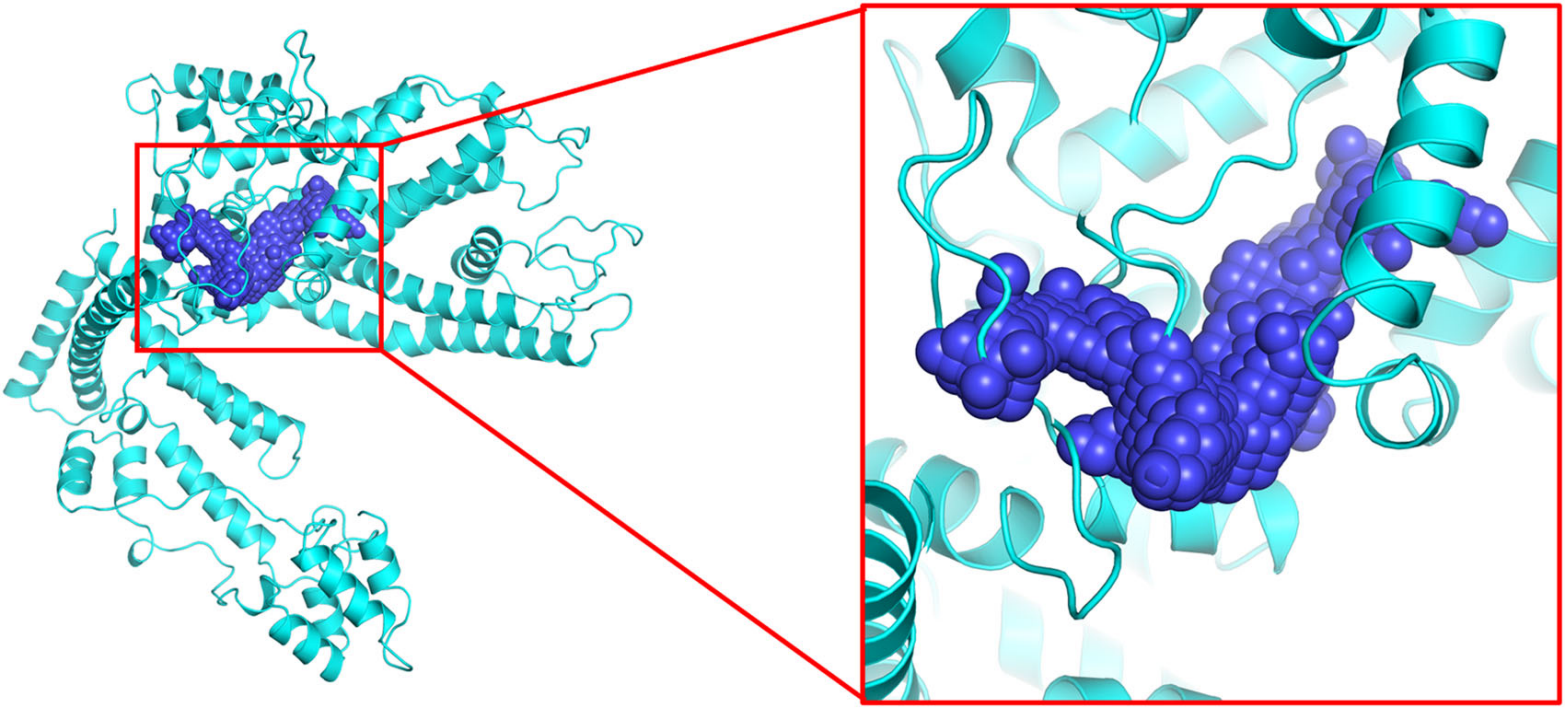
POCASA predicts protein active pockets of TRPC1. Purple beads represent potential active sites.

Swiss-Model was searched for the best template of TRPC1. It was found that the sequence of short transient receptor potential channel 5 (PDB ID: 6aei) is the most similar to that of TRPC1, and the similarity is 46.33%. Since reasonable conformations can be obtained with a model similarity above 30% (Bordoli et al. 2009), the modelling was performed with the short transient receptor potential channel 5 template to construct the TRPC1 protein 3D structure (Figure 1(A)). The Ramachandran plot was used to assess the quality of the model structure (Figure 1(B)). According to the Ramachandran plot, 91.44% of the TRPC1 amino acid residues are in the best region (green region). It is generally considered that a value larger than 90% indicates a reasonable protein model construction (Nagasundaram et al. 2016). Additionally, TRPC1 protein from homologous modelling has a certain influence on the accuracy of the model, and molecular dynamics simulation were performed using Gromacs 2020.1. As shown in Figure 1(C), the RMSD of the protein structure reached equilibrium after 80 ns during the simulation process, indicating that it was in a stable state at this time, and a stable conformation was randomly selected between 80 ns and 100 ns for subsequent docking experiments. The TRPC1 protein active pocket (Figure 2) was further predicted using POCASA to prepare for molecular docking.

### Preliminary screening results according to Lipinski’s rule of five and druggability

With *Salvia miltiorrhizae* as the keyword, 202 small molecule chemical components were retrieved from the TCMSP database. Then, a library of 202 compounds was constructed. The preliminary screening was performed according to Lipinski’s rule of five and other relevant screening conditions: molecular weight (MW) smaller than 500; octanol–water partition coefficient (Alog*P*) smaller than 5; hydrogen bond donors (Hdon) less than 5; hydrogen bond acceptors (Hacc) less than 10; oral bioavailability (OB) larger than 50% (due to the large number of chemical components of *Salvia miltiorrhizae*, the screening condition was set to OB larger than 50% in this study in order to make the focus of the study more clear); drug likeness (DL) larger than 0.18. The results showed that 20 compounds have relatively good characteristic parameters (Table 1).

**Table 1.** Twenty compounds that match initial screening conditions.

ID	Molecule name	MW	Alog*P*	Hdon	Hacc	OB (%)	DL
MOL001	Digallate	322.24	1.53	6	9	61.85	0.26
MOL002	2-(4-Hydroxy-3-methoxyphenyl)-5-(3-hydroxypropyl)-7-methoxy-3-benzofurancarboxaldehyde	356.40	3.58	2	6	62.78	0.40
MOL003	Formyltanshinone	290.28	3.36	0	4	73.44	0.42
MOL004	Przewalskin B	330.46	3.18	1	4	110.32	0.44
MOL005	Przewaquinone B	292.30	2.99	1	4	62.24	0.41
MOL006	Przewaquinone C	296.34	3.31	1	4	55.74	0.40
MOL007	Tanshinaldehyde	308.35	3.83	0	4	52.47	0.45
MOL008	Danshenol B	354.48	2.59	1	4	57.95	0.56
MOL009	Danshenol A	336.41	2.01	1	4	56.97	0.52
MOL010	Cryptotanshinone	296.39	3.44	0	3	52.34	0.40
MOL011	Danshenspiroketallactone	282.36	3.24	0	3	50.43	0.31
MOL012	Epidanshenspiroketallactone	284.38	2.37	0	3	68.27	0.31
MOL013	Isocryptotanshinone	296.39	3.59	0	3	54.98	0.39
MOL014	Miltionone II	312.39	2.14	1	4	71.03	0.44
MOL015	Neocryptotanshinone	314.41	3.01	2	4	52.49	0.32
MOL016	Prolithospermic acid	314.31	2.77	4	6	64.37	0.31
MOL017	Rosmarinic acid	360.34	2.69	5	8	109.38	0.35
MOL018	(*Z*)-3-[2-[(*E*)-2-(3,4-Dihydroxyphenyl)vinyl]-3,4-dihydroxy-phenyl]acrylic acid	314.31	2.82	5	6	88.54	0.26
MOL019	(6*S*)-6-Hydroxy-1-methyl-6-methylol-8,9-dihydro-7H-naphtho[8,7-g]benzofuran-10,11-quinone	312.34	2.42	2	5	75.39	0.46
MOL020	(6*S*)-6-(Hydroxymethyl)-1,6-dimethyl-8,9-dihydro-7H-naphtho[8,7-*g*]benzofuran-10,11-dione	310.37	3.57	1	4	65.26	0.45

MW: molecular weight; ALog*P*: partition coefficient; Hdon: number of hydrogen bond donors; Hacc: number of hydrogen bond acceptors; OB: oral bioavailability; DL: drug likeness.

### Molecular docking virtual screening score

**Figure 3.**
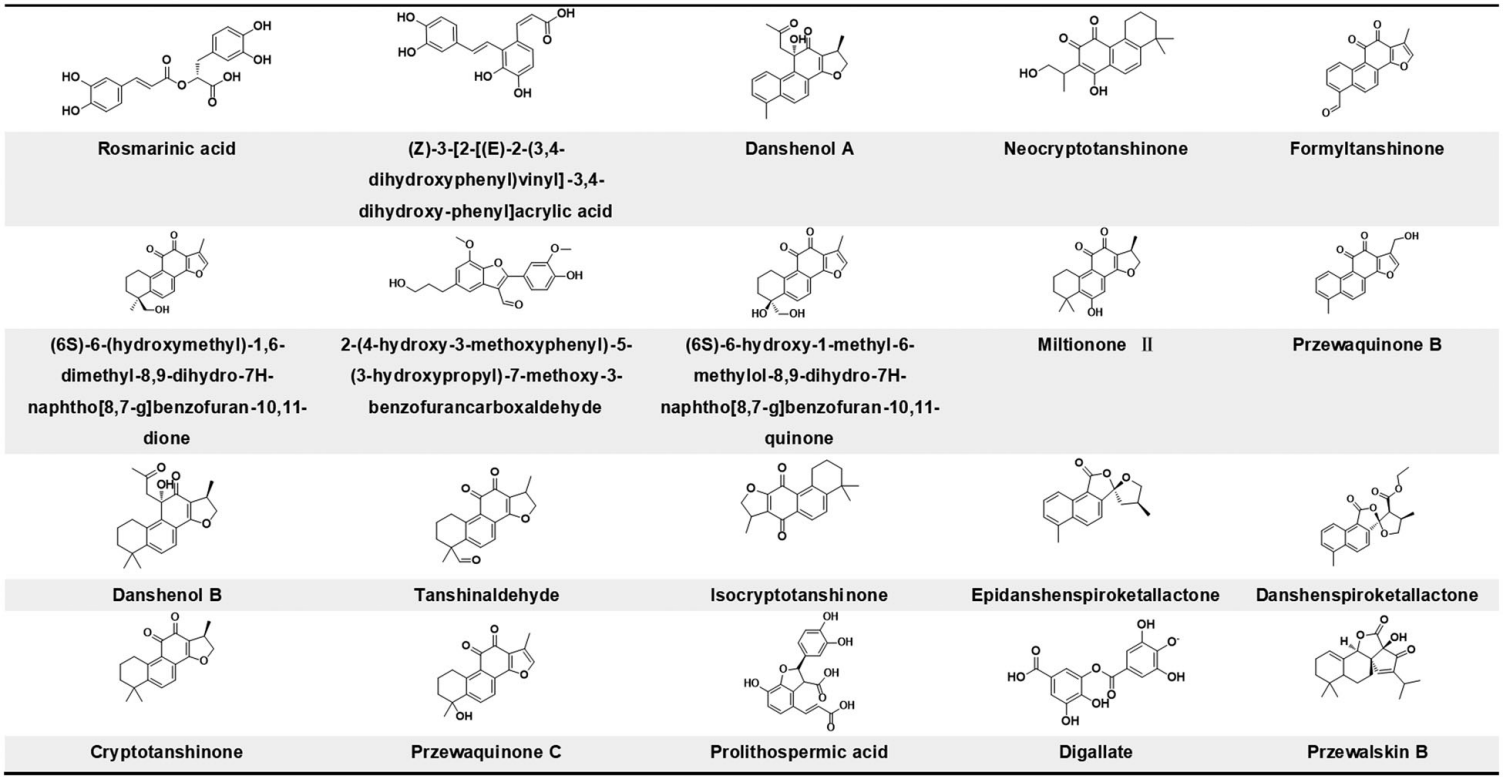
Structures of compounds sorted by docking scores results.

The binding energy of TRPC1 to 20 compounds was calculated by AutoDock Vina software, and the binding conformation was scored using a scoring function based on the shape matching and energy matching between the receptor and the ligand. The docking results of these compounds with receptors were ranked from highest to lowest, as shown in Table 2. The structures of all compounds are shown in Figure 3. RosA (ID: MOL017) was the compound that obtained the highest score.

**Table 2.** The results from high to low ranking of compounds.

ID	Name	Total score
MOL017	Rosmarinic acid	7.32
MOL018	(*Z*)-3-[2-[(*E*)-2-(3,4-Dihydroxyphenyl)vinyl]-3,4-dihydroxy-phenyl]acrylic acid	6.17
MOL009	Danshenol A	5.01
MOL015	Neocryptotanshinone	4.87
MOL003	Formyltanshinone	4.50
MOL020	(6*S*)-6-(Hydroxymethyl)-1,6-dimethyl-8,9-dihydro-7H-naphtho[8,7-*g*]benzofuran-10,11-dione	4.44
MOL002	2-(4-Hydroxy-3-methoxyphenyl)-5-(3-hydroxypropyl)-7-methoxy-3-benzofurancarboxaldehyde	4.44
MOL019	(6*S*)-6-Hydroxy-1-methyl-6-methylol-8,9-dihydro-7H-naphtho[8,7-*g*]benzofuran-10,11-quinone	4.41
MOL014	Miltionone II	4.38
MOL005	Przewaquinone B	4.29
MOL008	Danshenol B	4.24
MOL007	Tanshinaldehyde	3.89
MOL013	Isocryptotanshinone	3.88
MOL012	Epidanshenspiroketallactone	3.84
MOL011	Danshenspiroketallactone	3.84
MOL010	Cryptotanshinone	3.53
MOL006	Przewaquinone C	3.48
MOL016	Prolithospermic acid	3.13
MOL001	Digallate	3.12
MOL004	Przewalskin B	3.11

### The energy and space matching of the binding of RosA to the active cavity of TRPC1 protein

The free energy of RosA binding to the active cavity of TRPC1 protein was −8.4 kcal/mol, as calculated by AutoDock Vina software. The larger absolute value of the binding constant indicates less free energy required for the binding. It is generally considered that when the absolute value is greater than 7, the compound and the protein are more likely to bind (Nagasundaram et al. 2016).

### Stable binding of RosA to amino acid residues near the active site of TRPC1 protein

**Figure 4.**
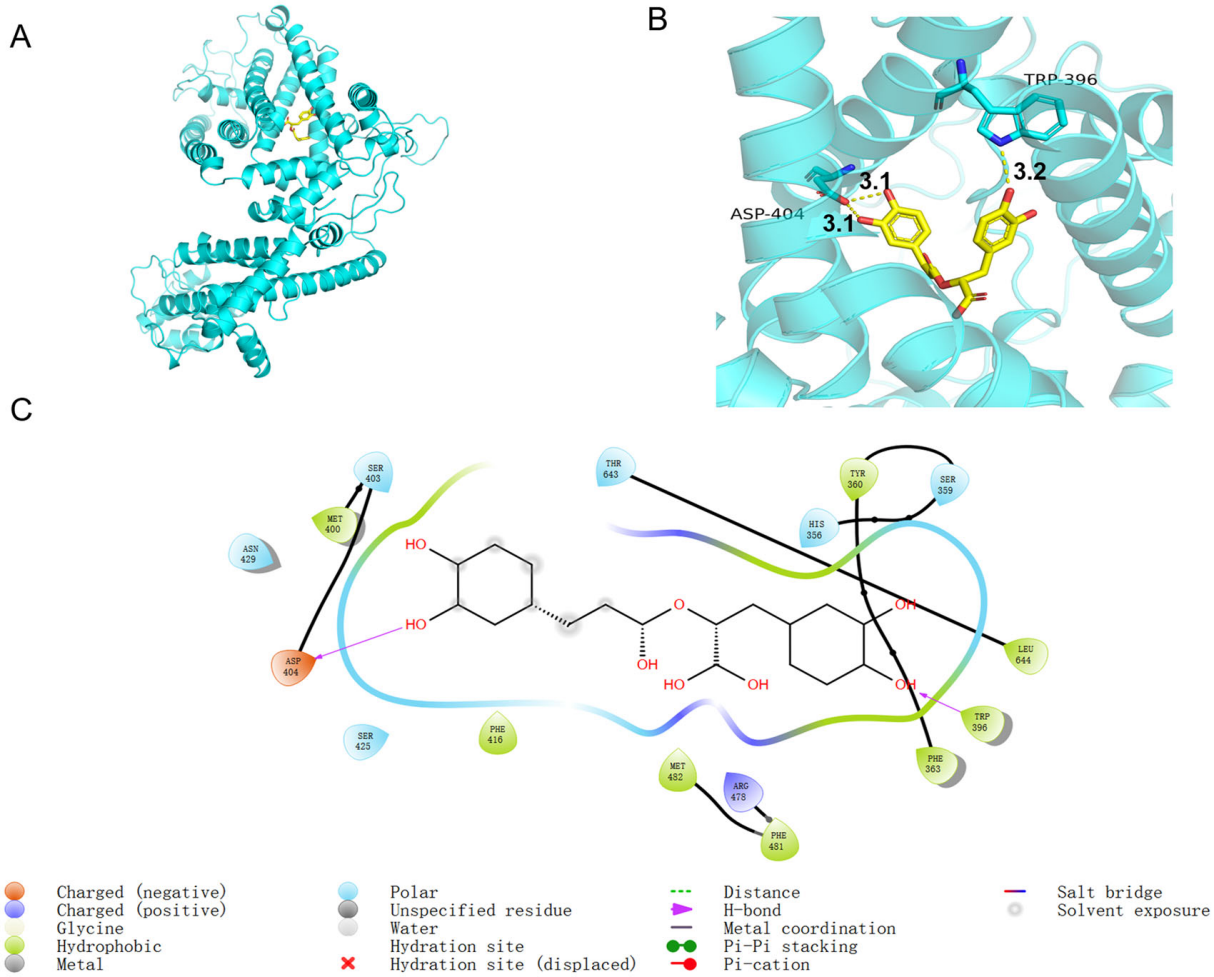
(A, B) Map of the interaction between RosA and TRPC1. Yellow dotted lines represent hydrogen bonds. ASP: aspartate; TRP: tryptophan. The lengths of the hydrogen bonds are 3.1 Å, 3.1 Å and 3.2 Å, respectively. (C) Two-dimensional map of the interaction between RosA and TRPC1.

The diagrams of the interaction between RosA and TRPC1 protein (Figure 4) show that the small molecules form three hydrogen bond interactions (the lengths of the hydrogen bonds are 3.1 Å, 3.1 Å and 3.2 Å) with two amino acids, namely, ASP (aspartate) 404 and TRP (tryptophan) 396, near the active site, and form a salt bridge with ARG 478. The polar interaction between the hydrogen bond and the salt bridge provides a considerable electrostatic force for compound binding. The small molecules form a π–π conjugation stacking interaction with PHE (phenylalanine) 481, providing a strong van der Waals force for compound binding. These interaction forces contribute to the stable binding of RosA to the TRPC1 protein active site.

### Results of affinity assay for RosA and TRPC1

**Figure 5.**
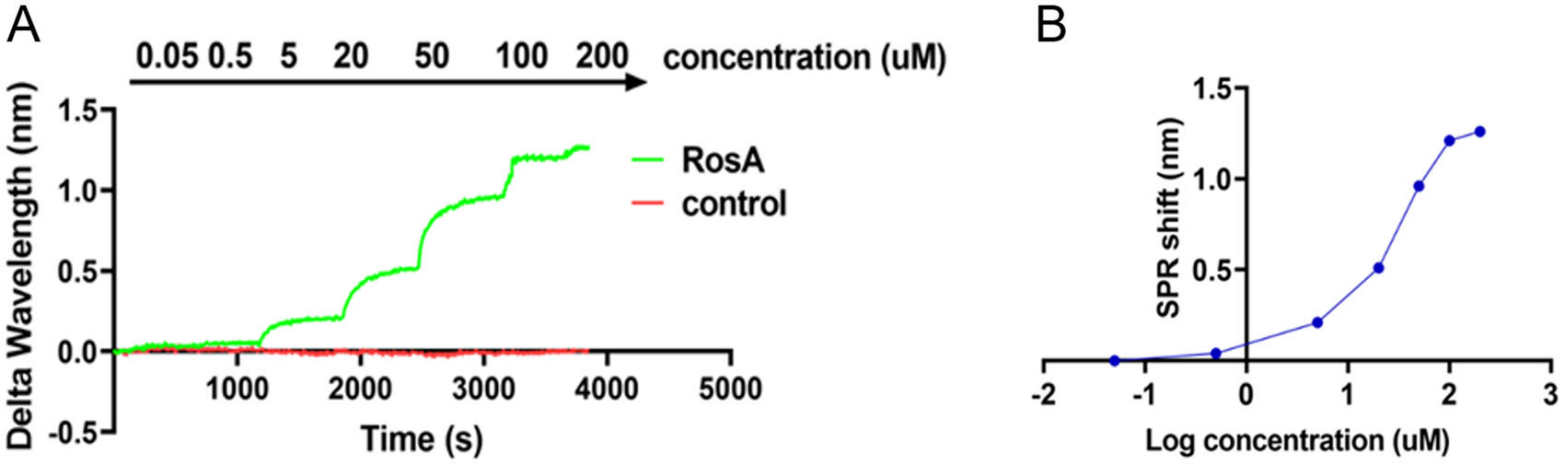
The SPR binding curve of RosA and TRPC1. (A) A single sample detection experiment was carried out on the RosA solution with concentration gradient. (B) The equilibrium dissociation constant (KD) between RosA and TRPC1 was 1.27 μM.

SPR assay was used to validate whether RosA binds to TRPC1 protein. The purity and structure of RosA were validated by HPLC and 1H NMR, respectively, and RosA could be used for SPR experiments. Equilibrium dissociation constant (KD) value is an indicator for evaluating compound–protein interactions in SPR experiments, and a smaller KD indicates a greater affinity. The affinity of RosA to TRPC1 was evaluated by measuring KD values and was compared with that of TRPC1 antibody and inhibitor SKF96365 to TRPC1. The results showed that the KD value for the affinity of RosA to TRPC1 was 1.27 µM, while the KD values for the affinity of TRPC1 antibody and SKF96365 to TRPC1 were 0.05 µM and 200 µM, respectively, indicating that RosA can directly bind to TRPC1 (Figure 5(A,B)).

### Reduction of TRPC1 protein levels in HL-1 cells by RosA after OGD/R

**Figure 6.**
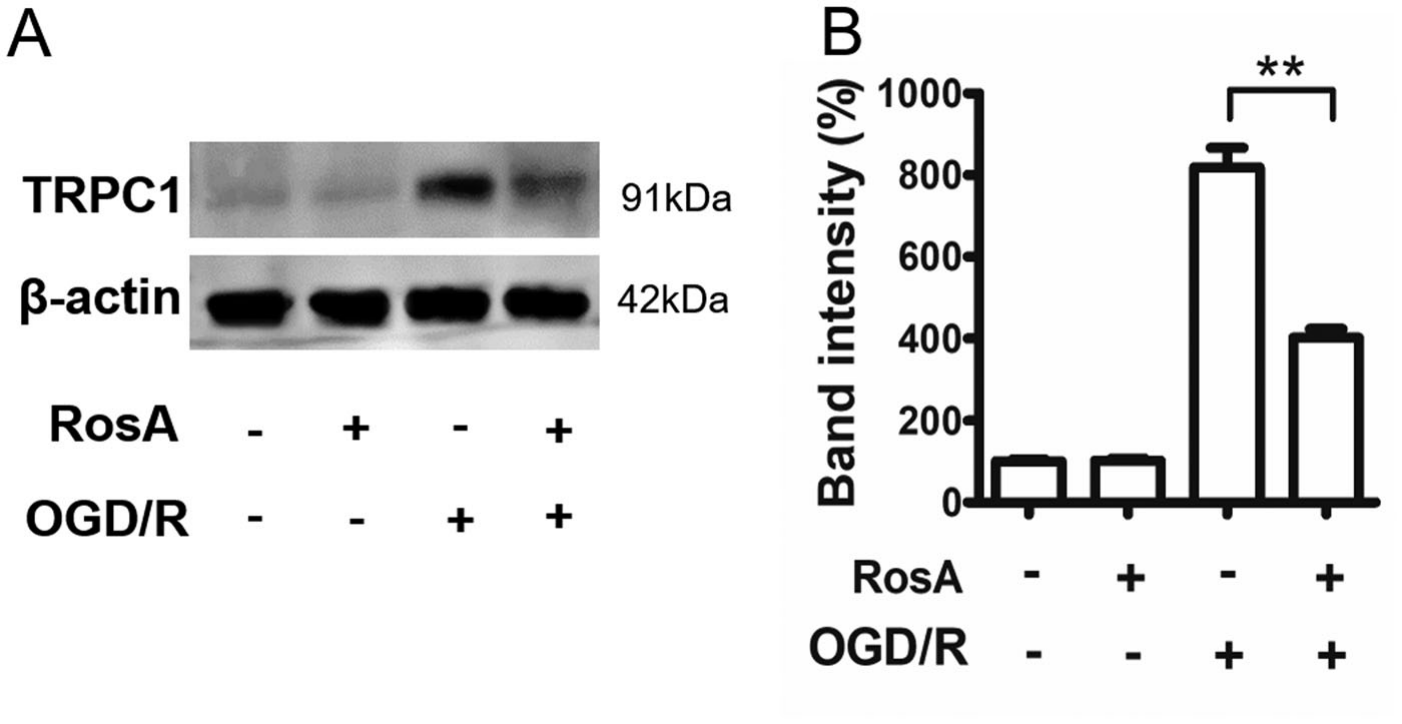
RosA inhibits TRPC1 protein levels after OGD/R in HL-1 cell. HL-1 cells were treated with 50 μM RosA, and then were injured by OGD/R (9 h/6 h). (A) The level of TRPC1 protein in cells was detected by western blot method. RosA inhibits TRPC1 protein levels after OGD/R in HL-1 cells. (B) Quantitative statistical results of TRPC1 protein expression. *N* = 3, **p< 0.01.

Western blot was used to detect TRPC1 protein levels after OGD/R in HL-1 cells. TRPC1 protein levels were significantly increased after OGD/R injury. RosA (50 μM) could decrease TRPC1 protein levels after OGD/R in HL-1 cells (401.7%±22.2% vs. 818.8%±48.7%), but had no effect on that in the normoxic group (Figure 6(A,B)). The above experimental results show that RosA reduces TRPC1 protein levels after OGD/R in HL-1 cells.

### Inhibition of TRPC1-mediated Ca^2+^ influx in HKE293 cells by RosA

**Figure 7.**
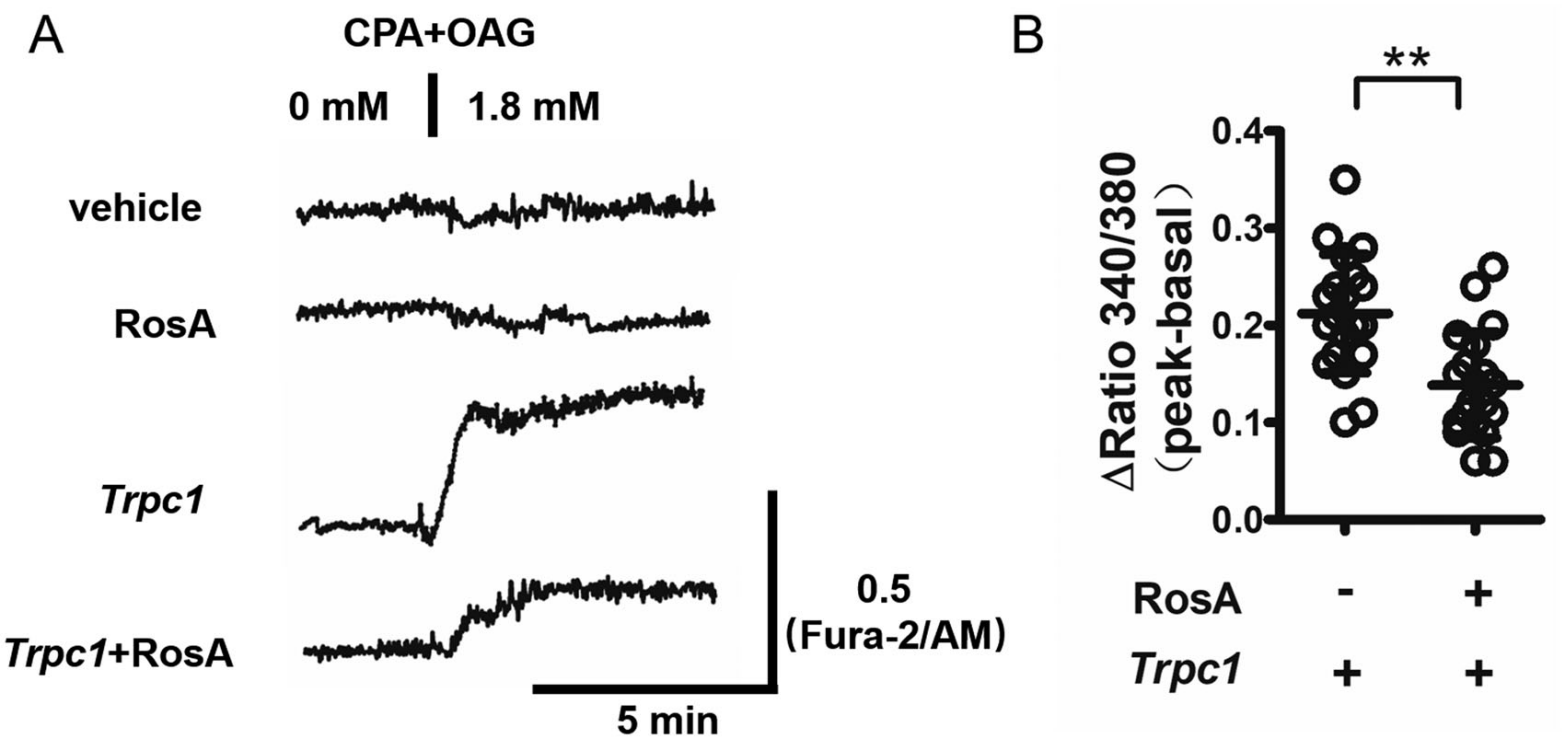
RosA inhibits TRPC1-mediated Ca^2+^ influx in HEK293 cells. HEK293 cells were transfected with plasmid vector pcDNA3.1 to *Trpc1* for 6 h and then cultured for 24 h. Cells were treated with 50 μM RosA for 6 h and then Ca^2+^ influx was detected. (A) Ca^2+^ influx was significantly increased after overexpressed Trpc1 HEK293 cells, while RosA inhibited TRPC1-mediated Ca^2+^ influx in HEK293 cells. (B) The difference in Fura-2 340/380 ratio was calculated and statistically analysed. *N* = 20, **p< 0.01.

The effect of RosA on TRPC1-mediated Ca^2+^ influx was examined with the Fura-2/AM method. It was found that overexpression of *Trpc1* in HEK293 cells significantly increased Ca^2+^ influx, and RosA inhibited Ca^2+^ influx mediated by *Trpc1* overexpression (0.14 ± 0.05 vs. 0.21 ± 0.06) (Figure 7(A,B)).

## Discussion

*Salvia miltiorrhizae* is a common traditional Chinese herb with an application history of nearly two thousand years. *Compendium of Materia Medica* records that *Salvia miltiorrhizae* can promote blood circulation to remove the obstruction. It is believed that *Salvia miltiorrhizae* has the same effect as four substances decoction, whose ingredients include *Paeonia lactiflora* Pall. (Paeoniaceae), *Ligusticum chuanxiong* Hort. (Apiaceae), *Angelica sinensis* (Oliv.) Diels (Apiaceae) and *Rehmanniae* (Plantaginaceae) (MEIm et al. 2019). A variety of drugs have been successfully developed for clinical application from *Salvia miltiorrhizae*. They have been used to treat cardiovascular system diseases (Orgah et al. 2020), skin diseases (Hu and Ren 1998), liver and kidney diseases (Cao et al. 2017), etc., and have achieved good effects. In this study, we found the RosA from the *Salvia miltiorrhizae* acting on the novel target TRPC1 based on the ‘homology modelling–virtual screening–molecular docking–affinity assay–activity evaluation’ method.

First, the TCMSP database was searched for *Salvia miltiorrhizae* compounds, and a total of 202 small molecule chemical components were retrieved. The preliminary screening was performed according to Lipinski’s rule of five and other relevant screening conditions. It was found that 20 compounds (9.9%) have relatively good characteristic parameters. Molecular docking was performed with AutoDock Vina software, and it was found that RosA had the highest score. The free energy for the binding of RosA to TRPC1 protein was found to be −8.4 kcal/mol. A larger absolute value of the binding constant indicates less free energy required for the compound binding. It is generally considered that when the absolute value is greater than 7, the small molecules and the protein are more likely to bind (Nagasundaram et al. 2016). It was indicated that RosA and the active pocket of TRPC1 have good complementary spatial and electrical features, which make it easy to form a stable binding conformation. The analysis of the docking pattern of RosA and TRPC1 protein revealed that the small molecules formed three hydrogen bond interactions with two amino acids, namely, ASP 404 and TRP 396, near the active site and formed a salt bridge with ARG 478. The polar interaction of the hydrogen bond and the salt bridge provided considerable electrostatic force for the binding of RosA to TRPC1. Additionally, small molecules formed π–π conjugation stacking interaction with PHE 481, providing a strong van der Waals force for compound binding. These interaction forces contribute to the stable binding of RosA to amino acid residues 350–650 near the active site of TRPC1 protein, and the affected sequence of this residue will probably impair the function of TRPC1 at the cell membrane and may also lead to the inhibition of Ca^2+^ influx mediated by TRPC1.

Molecular docking technology still has many shortcomings and limitations, and the calculation results cannot replace the experimental data. It needs to be combined with other methods (Tie et al. 2012). To further determine whether RosA interacted with TRPC1 protein, we examined the affinity of RosA to TRPC1 using SPR and found the binding KD was 1.27 µM (0.05 µM for the affinity between TRPC1 antibody and TRPC1, 200 µM for the affinity between SKF96365 and TRPC1). Small KD indicates a large affinity of the ligand to its target protein. Based on this approach, we found that the exact active component in *Salvia miltiorrhizae* was RosA.

RosA is a common natural water-soluble polyphenolic compound. It is obtained from the condensation of one molecule of tanshinone and one molecule of caffeic acid and is an oligomeric homologue of caffeic acid. It is widely distributed in herbal medicines, mainly found in plants of the Labiatae family (*Salvia miltiorrhizae*, *Rosmarinus officinalis*, etc.) and the Boraginaceae family (*Arnebiae Radix*) (Cai et al. 2019; Cardullo et al. 2019). Rosmarinic acid was named after *Rosmarinus officinalis* since it was first isolated from this plant in 1958. Since the 1990s, water-soluble active components represented by RosA and salvianolic acid B have become a hot topic in the research of *Salvia miltiorrhizae*. RosA, the metabolic intermediate of *Salvia miltiorrhizae*, is the core precursor for the synthesis of salvianolic acid B. In addition, RosA can be metabolized in animals and degraded to active components, including tanshinone and caffeic acid (Sun 2008). It has been found that the content of RosA in *Salvia miltiorrhizae* is 1.091 mg/g (Zheng 2010). The main active ingredient of salvianolate for injection (DUOPUSAI^®^) is polyphenolic acid, of which RosA, salvianolic acids B, D, E, and lithospermic acid account for more than 80% (Zhou and Yan 2017). It has also been found that the main pharmacodynamic substances of Dan Hong Injection [*Salvia miltiorrhizae* – *Carthamus tinctorius* L. (Compositae)] contain RosA, the content of which is 202.11 μg/mL, second to that of salvianolic acid B (Zhou 2013). Additionally, a study has identified that the *in vivo* therapeutic material bases of *Salvia miltiorrhizae*–*Dalbergia odorifera* are RosA, salvianolic acid A, tanshinone isopropanol ester and caffeic acid isopropanol ester (Yang 2018). All the above studies confirmed that RosA is one of the main active components of *Salvia miltiorrhizae*, which is consistent with the result of ‘virtual screening–molecular docking–affinity assay’ in the present study.

Further, *in vitro* activity tests revealed that RosA could reduce TRPC1 protein levels after OGD/R in HL-1 cells; it also inhibited TRPC1-mediated Ca^2+^ influx in HEK293 cells, indicating that RosA has better *in vitro* activity and that RosA may not only affect the function of TRPC1 but also influence its expression by binding to it.

## Conclusions

Searching active compounds from TCM has re-emerged as a hot topic in recent years. In this study, we found the exact active component-RosA-acting on the TRPC1 target from the *Salvia miltiorrhizae* using ‘homology modelling–virtual screening–molecular docking–affinity assay–activity evaluation’, a new TCM drug discovery and evaluation model. Although this research needs to be complemented by further pharmacological experiments, we speculate that RosA may be a promising candidate for future clinical applications.
